# Endoplasmic reticulum membrane protein MoScs2 is important for asexual development and pathogenesis of *Magnaporthe oryzae*

**DOI:** 10.3389/fmicb.2022.906784

**Published:** 2022-08-04

**Authors:** Jun Zhang, Xuehang Chen, Zifeng Yang, Huxiao Xu, Shuning Weng, Zonghua Wang, Wei Tang

**Affiliations:** ^1^State Key Laboratory of Ecological Pest Control for Fujian and Taiwan Crops, Ministerial and Provincial Joint Innovation Centre for Safety Production of Cross-Strait Crops, College of Plant Protection, Fujian Agriculture and Forestry University, Fuzhou, China; ^2^Institute of Oceanography, Minjiang University, Fuzhou, China; ^3^Fujian Key Laboratory for Monitoring and Integrated Management of Crop Pests, Fuzhou, China

**Keywords:** *Magnaporthe oryzae*, ER-PM, tail-anchored protein, pathogenesis, asexual development

## Abstract

Most secretory proteins are folded and modified in the endoplasmic reticulum (ER). In *Saccharomyces cerevisiae*, the absence of Scs2 protein will lead to the separation of the endoplasmic reticulum and plasma membrane, resulting in endoplasmic reticulum dysfunction, but its function is not clear in rice blast fungus or even filamentous fungus. In this study, we report the identification and characterization of *MoSCS2* in the pathogenesis of the rice blast fungus *Magnaporthe oryzae*. Protein subcellular localization showed that MoSCS2 is mainly localized in the endoplasmic reticulum. Compared to the wild-type strain Guy11, the deletion mutant Δ*Moscs2* showed a significant reduction in growth and conidiation. *MoSCS2* deficiency also resulted in abnormal conidial morphology and septum formation. The Δ*Moscs2* mutant shows delayed appressorium formation, and the appressorium of Δ*Moscs2* mutant could not form huge turgor pressure to penetrate the host epidermal cell wall. Pathogenicity and plant leave infection assays showed that knockout of *MoSCS2* significantly inhibited the expansion of the invasive hyphae in host cells, ultimately leading to the decline of pathogenicity. Moreover, *MoSCS2* gene is also involved in the regulation of cell wall and endoplasmic reticulum stress response. In conclusion, *MoSCS2* plays an important role in the growth, asexual production, conidia morphogenesis, infection-related morphogenesis and pathogenicity of *M. oryzae.*

## Introduction

*Magnaporthe oryzae*, an intriguing ascomycete fungal pathogen causes the most devastating rice blast disease (Talbot, 2003). It spreads rapidly between hosts by wind and rain and impacts rice production worldwide, especially in the changing climate (Pringle and Taylor, 2002). It has become a model organism for studying the interaction between pathogens on plants and the plants they infect (Ebbole, 2007; Dean et al., 2012). The fungus produces pyriform conidia, which is important for fungal pathogenesis, partially because conidia are the primary inoculums and facilitate disease dissemination in the host (Howard et al., 1991). Conidia are produced by the apex of the conidiophores where two round mitotic divisions and the formation of two septa. Finally, the conidia form a three-celled structure by nuclear migration and positioning. During the appropriate environment condition, germination and differentiation of the conidium result in a specialized infection structure on the hydrophobic surface, appressorium (Dean, 1997). Interestingly, the appressorium can generate an enormous turgor pressure to mechanically penetrate plant tissues (Oses-Ruiz et al., 2017). Subsequently, invasive hyphae (IH) are produced by the penetration peg that can rapidly penetrate the cuticle and underlying plant cells (Yi and Valent, 2013). After colonization for 1 week, a large number of infection diseases appear on the leaf surface, where conidia are produced and enter new invasive breeding (Howard and Valent, 1996). To promote infection, *M. oryzae* secretes a large number of effector proteins during rice–pathogen interaction to inhibit plant defense response (Fernandez and Orth, 2018). There are two different secretory pathways of effector proteins during infection. Secretion of apoplastic effectors takes place within the traditional ER-Golgi secretory pathway, in contrast, the exocyst complex is responsible for delivering cytoplasmic effectors (Giraldo et al., 2013). In eukaryotic cells, proteins secreted by ribosomes are processed and modified within the ER (Liu et al., 2014). Upon reaching the Golgi apparatus, mature proteins are packed into secretory vesicles, then they can release their contents into the extracellular space by fusion with the cytoplasmic membrane (Cui et al., 2015). As is known to us the ER contacts numerous organelles. There is a special connection between the ER and the plasma membrane (PM), which is called ER-PM (Stefan et al., 2013). The ER-PM junction plays an important role in many physiological processes, such as lipid metabolism and control of calcium (Ca^2+^) dynamics (Spang, 2018). In yeast cells, the cortical ER (cER) is extensively associated with PM, and this association requires several tethering proteins that staple them together (West et al., 2011).

There are at least six proteins that act as tethers contribute to ER–PM contact sites: two VAMP-associated proteins (Ssc2/22), three extended synaptotagmins (*Tcb1*/*2*/*3*), and the putative ion channel (*Ist2*; Loewen and Levine, 2005; Stefan et al., 2011; Manford et al., 2012). Ist2 is related to the TMEM16-anoctamin family of ion channels and phospholipid scramblases, the deletion of *Ist2* results in a significantly lower proportion of cER structures, while the overexpression of *Ist2* results in the increase of ER-PM contact sites (Kunzelmann et al., 2016). Scs2 and Scs22 are homologs of the yeast vesicle-associated membrane protein (VAMP)-associated protein (VAP; Kato et al., 2017). They were anchored in the ER through the C-terminal (transmembrane domain) and contained the MSP (Major SPERM Protein) domain at the N-terminal (Quon et al., 2018). The MSP domain of Scs2/22 can bind to plasma membrane proteins containing FFAT (two phenylalanines in an acidic tract) or FFAT-like motifs (Loewen and Levine, 2005; Manford et al., 2012), In yeast cells, Scs2/22 are associated with cER inheritance, deletion of Scs2/22 will cause a loss of cER associated with the PM (Loewen et al., 2007). Scs2/22 also has a PH domain that interacts with phosphoinositides at the PM (Kaiser et al., 2005). At the ER-PM contact sites, Scs2 interacts with the oxysterol-binding homology protein Osh3 to activate Sac1 which is located in ER. The interaction between Scs2 and Septin Shs1 can form an ER diffusion barrier to prevent intact ER proteins from diffusing between the ERs of the mother and the daughter cells (Luedeke et al., 2005; Chao et al., 2014). Tcb1/2/3 bind to the PM by means of lipid-binding C2 domains, while TCB1/2/3 also has an SMP domain, which participates in the exchange of phospholipids and diglycerol esters between the PM and ER (Giordano et al., 2013). Tcb1/2/3 was homologous to the extended synaptotagmin-like proteins E-syt1/2/3 (Min et al., 2007). The absence of all six proteins results in a massive reduction in ER-PM contacts and morphological changes in ER (Manford et al., 2012). Meanwhile, misregulation of phosphoinositol signaling at PM occurs in cells lacking the ER-PM adaptor protein (Zhang et al., 2012), and the activation of unfolded protein response is constitutive in the ER (Luedeke et al., 2005). In conclusion, ER-PM contact sites play an important role in cell signal transduction organelle morphology and endoplasmic reticulum function.

Previous studies have demonstrated that MCS (Membrane Contact Sites) between ER and PM play an important role in eukaryotic cells, and this connection is critical for the transport of substances such as protein lipids and ions between ER and PM (Mentlak et al., 2012), it can provide a platform for material exchange and signal transduction between organelles (Stefan et al., 2013). The cER and PM make extensive contacts, and the ER-localized PI phosphatase Sac1 reversely regulated the level of phosphatidylinositol-4-phosphate (PI4P; Stefan et al., 2011). It is observed in yeast cells that Scs2/ Scs22 contribute to the ER-PM junction and Sac1-mediated PI4P turnover (Loewen et al., 2007; Stefan et al., 2011).

In this study, we identified Scs2 orthologous protein in *M. oryzae.* To investigate the importance of Scs2 in *M. oryzae*, we analyzed the functions of Scs2 in the different developmental stages. Based on our studies, we demonstrate that Scs2 is located in the ER and plays a crucial role in vegetative growth, asexual reproduction infection-related morphogenesis and pathogenicity of *M. oryzae*.

## Materials and methods

### Strains and culture conditions

The *M. oryzae* strain Guy11 was used as WT for transformation in this study. All strains were cultured on CM agar plates (CM: 10 g D-glucose, 2 g peptone, 1 g yeast extract, 1 g casamino acids, 50 ml 20 × nitrate salts, 1 ml trace elements, 1 ml vitamin solution, 15 g agar, add distilled water to 1 l) at 28°C (Talbot et al., 1993). Liquid CM medium was used to harvest the mycelia for protoplast preparation, genomic DNA, RNA and protein extraction.

The protoplast-mediated transformation of *M. oryzae* was performed for gene deletion and complementation assays by using hygromycin B and bleomycin as a selective marker as described (Sweigard et al., 1992). Hygromycin B (250 mg/ml, Calbiochem, La Jolla, CA) or bleomycin (200 mg/ml, Invitrogen, Carlsbad, CA) was used for transformant selection on TB3 medium (3 g of yeast extract, 3 g of casamino acids, 200 g of sucrose, 7.5 g of agar in 1 l of distilled water).

### Targeted gene deletion and complementation

The gene-deletion mutants were generated using the standard one-step gene replacement strategy as described (Tang et al., 2015; Pan et al., 2019). Two 1.0 kb of sequences flanking the targeted gene was PCR amplified from *M. oryzae* genomic DNA using the primer pairs (Supplementary Table S1) respectively; the hygromycin phosphotransferase (*hph*) cassette were PCR amplified from pCX62 using the primer pair HYG F(F)/HYG R(R; Supplementary Table S1). The double-joint PCR approach was used to generate the gene replacement construct for the *MoSCS2* gene which contained the flanking sequences and *hph* cassette for each gene were transformed into protoplasts of the WT Guy11 (Yu et al., 2004). Putative mutants were firstly screened by PCR and further confirmed by Southern blotting analysis (Supplementary Figure S1; Supplementary Table S1). One mutant strain for either gene disruption (Δ*Moscs2#3*) was randomly selected for further analysis (Supplementary Figures S1C,D). The complement fragments, which contain the entire *MoSCS2* gene and the native promoter regions, were amplified by PCR with primers (Supplementary Table S1) and inserted into pYF11 (Bleomycin resistance) to generate *MoSCS2-GFP* or *MoSCS2*^Δ^*^TMD^-GFP* fusion constructs which were used to complement the respective mutant strains. Transformants expressing the constructs were identified by PCR and confirmed by fluorescent microscopy.

### Assays for vegetative growth, conidiation, and appressorium formation

Small agar blocks were cut from the edge of 4-day-old cultures and placed onto fresh media (CM and SDC) for culturing in the dark at 28°C for 7 days, and colony diameter was measured by ruler before photographing. To assay for defects in ER stress responses, the growth rate was measured with cultures grown on CM with 2 mM DTT or 0.2 μg/ml TM. For conidiation, strain blocks were maintained on straw decoction and corn (SDC: 100 g of straw, 40 g of corn powder, 15 g of agar in 1 l of distilled water) agar media at 28°C for 7 days in the dark followed by 3 days continuous illumination under fluorescent light. Calcofluor White (CFW) staining was performed by using fluorescent brightener 28 (10 μg/ml, Sigma-Aldrich) for the microscopy of conidia and viewed under the fluorescence microscope. Appressorium formation on artificial hydrophobic surface and infection on barley epidermal cells were measured as described previously (Tang et al., 2019). For appressorium induction on rice leaves, conidial suspension (5 × 10^4^ spores per milliliter) was dropped on rice leaves and directly observed under microscopy.

### Pathogenicity and plant leave infection assays

Plant infection and injection assays were performed as described (Zhang and Xu, 2014). Conidia were resuspended to a concentration of 5 × 10^4^ spores per milliliter in 5 ml of 0.2% (w/v) gelatine solution. For detached barley leaves inoculation, 5-day-old barley (*Hordeum vulgare* cv. Golden Promise) leaves were cut and laid into 15 cm dishes under humid conditions, and 20 μl of the conidial suspension was placed onto each inoculation site of barley leaves followed by incubation at 28°C for 5 days and photographed. For spray inoculation on rice, a conidial suspension was sprayed onto 2-week-old seedlings of rice (*O. sativa* cv. CO39). For injected inoculation, conidial suspension was injected into the rice sheath by using 1 ml syringe. Inoculated plants were kept in a growth chamber at 25°C with 90% humidity and in the dark for the first 24 h, followed by a 12-h/12-h light/dark cycle for 7 days to examine the lesion formation. Host-derived ROS was detected by staining with DAB (3,3-diaminobenzidine, D-8001, Sigma-Aldrich, United States) as described (Wang et al., 2019). For microscopic observation of hyphal expansion in plant cells, the same concentration of conidial suspension was injected into the detached rice sheaths followed by incubation at 28°C for 36 h, and rice sheaths were observed and photographed under a light microscope (Tang et al., 2019).

### Protein subcellular localization

A Nikon A1 plus confocal microscope (Nikon, Tokyo, Japan) was used to observe fluorescent light of transformants expressing MoScs2-GFP or MoScs2^Δ^^TMD^-GFP. The emission wavelength and excitation wavelength is 525.0 nm and 488.0 nm for GFP fluorescence.

### Stress response

Mycelia plugs were placed onto CM agar plates with 2 mM DTT, 0.2 μg/ml TM, 400 μg/ml CFW, 400 μg/ml CR, 0.005% SDS and cultured in the dark at 28°C for 7 days to determine their effects on fungal growth. The inhibition rate was determined by the percent decrease in the colony diameter. The experiment was repeated three times with three replicates each time.

## Results

### Identification and characterization of MoSCS2 in *Magnaporthe oryzae*

Scs2 orthologs are well conserved in fungi. We performed a BLASTP search of the *M. oryzae* genome^1^ using the sequence of Scs2/Scs22 from *Saccharomyces cerevisiae* as a query and named *MoSCS2* (MGG_06183). *MoSCS2* is predicted to encode a 285-amino-acid (aa) protein, respectively. Domain prediction reveals that MoSCS2 possesses a motile sperm domain (108 aa) at the N-terminus and a transmembrane domain (TMD; 20 aa) at the C-terminus (Supplementary Figure S1A).

### Targeted gene deletion and complementation of MoSCS2

To test the function of *MoSCS2*, we generated a construct for gene replacement and transformed into the wild-type (WT) Guy11. At least five independent *MoSCS2* deletion mutants with identical phenotypes were verified by PCR and confirmed by Southern blot analysis using gene-specific primers and probes (Supplementary Figure S1; Supplementary Table S1). Therefore, one mutant strain (Δ*Moscs2#3*) was randomly selected for further analysis (Supplementary Figures S1C,D). To ascertain that the observed phenotypes of the gene disruption mutants were due to the deletion of the *MoSCS2* gene, the complementation of transformants was generated by transforming the native promoter-driven MoScs2-GFP fusion constructs into the corresponding mutant. The complemented strains with GFP signals were achieved and recovered all the phenotypic defects. One of which was randomly selected for further study.

### MoScs2 is an ER-localized protein in *Magnaporthe oryzae*

**Figure 1:**
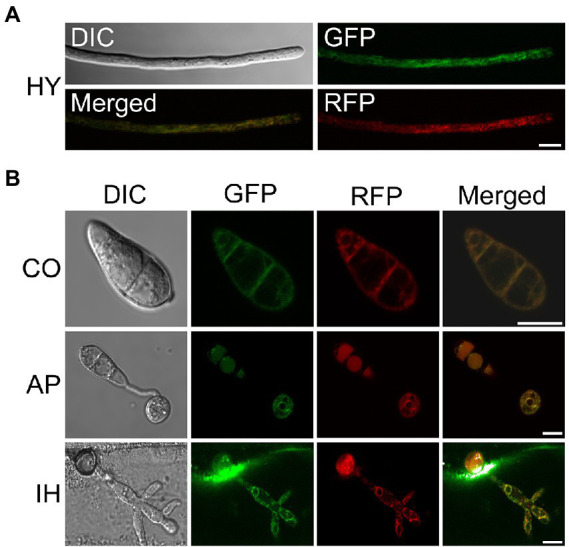
Subcellular localization of MoScs2 at different developmental stages of *M. oryzae*. **(A)** Hyphae (HY) co-localization of MoScs2-GFP together with the MoLhs1-RFP endoplasmic reticulum marker. **(B)** Conidia (CO), appressorium (AP) and invasive hyphae (IH) co-localization of MoScs2-GFP together with the MoLhs1-RFP endoplasmic reticulum marker. Localization of MoScs2-GFP were examined by Nikon laser confocal. Scale bar = 10  μm. DIC, Differential interference contrast.

To further clarify the localization characteristics of *MoSCS2* in different growth and development stages of *M. oryzae*, we constructed the GFP fusion protein expression vector of *MoSCS2* (pYF11::*MoSCS2*). We then co-expressed the ER-targeted MoLhs1-RFP in the fluorescent strain expressing MoScs2-GFP strain. Co-localization between MoScs2-GFP and MoLhs1-RFP supported our assumption that *MoSCS2* can be expressed in vegetative mycelia, conidia, appressorium, and infection mycelia (Figures 1A,B). These results suggested that MoScs2 was localized in the ER during each growth and development stage of *M. oryzae*.

### MoSCS2 is important for hyphae growth, conidiation and conidia morphology

**Figure 2:**
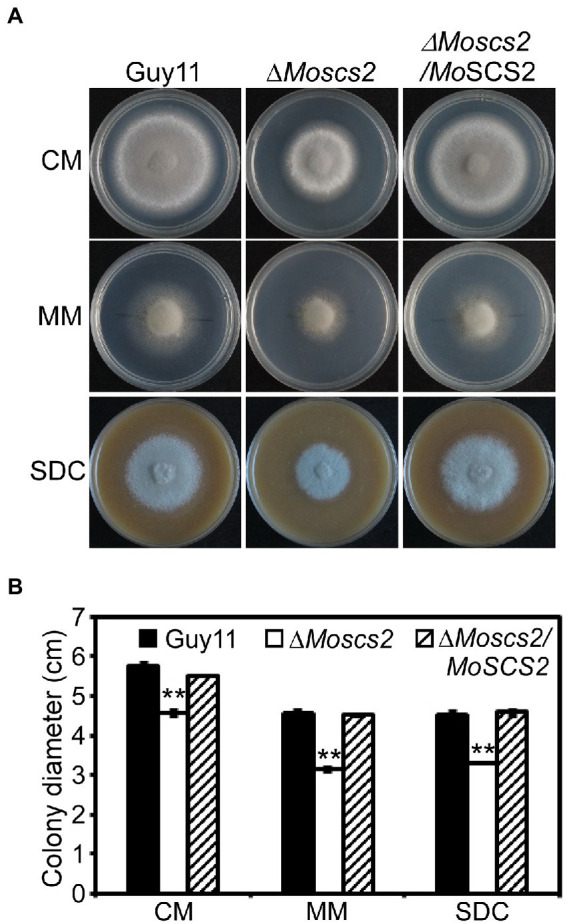
Effects of MoScs2 on hyphal growth. **(A)** Colony morphology and diameter of WT, Δ*Moscs2*, and the complemented strain cultured on CM, SDC, and MM. Photographs were taken after 7 days of incubation. **(B)** Statistical representation of average growth characteristics of the Δ*Moscs2* mutant compared to Guy11 and the complemented strain. Data used for statistical computation were obtained from three independent biological experiments with each time four replicates each time. Experiments were repeated with similar results. Asterisks represent a significant difference between Guy11 and the Δ*Moscs2* strain (Duncan’s new multiple range test, *p* < 0.01).

To investigate the role of MoScs2 in vegetative growth, analyses were carried out in *M. oryzae* to determine the effects of disrupting *MoSCS2* on growth and morphology, and the growth phenotype was compared with WT and its corresponding complemented strains. After incubation at 28°C for 7 days, a recognizable change in colony morphology was observed in Δ*Moscs2* mutant colonies. As compared to the WT, the Δ*Moscs2* developed significantly slower, and a reduced number of aerial hyphae was evident (Figure 2). These results indicated that *MoSCS2* has an important role in vegetative hyphal growth.

**Figure 3:**
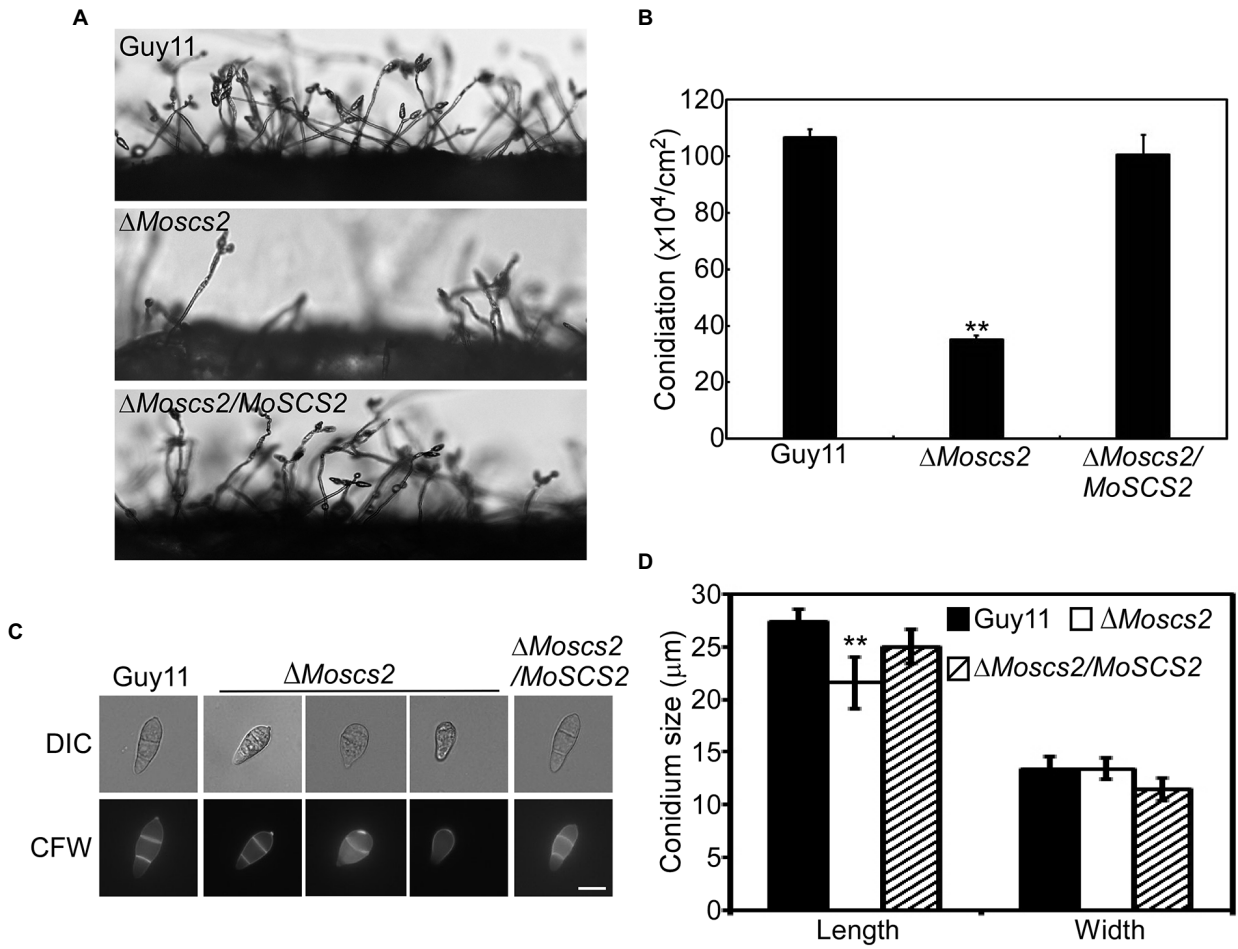
MoScs2 is important for asexual development. **(A)** Conidia formation was observed under a light microscope after 24 h incubation at room temperature after induction of conidiation on coverslips. **(B)** Statistical representation of the conidiation characteristics of the Δ*Moscs2* mutant, the WT and the complemented strain. **(C)** Conidium shape comparison. The conidia of the indicated strains were stained with Calcofluor white (CFW) and photographed. **(D)** Statistical analysis of the conidium size of the indicated strains. Error bars represent SD of three replicates and asterisks represent a significant difference between Guy11 and the Δ*Moscs2* strain (Duncan’s new multiple range test, *p* < 0.01). Scale bar = 10 μm.

To investigate the role of *MoSCS2* in asexual development, quantifying conidium production of the corresponding strains on SDC media was carried out as well. Compared with the WT and Δ*Moscs2/MoSCS2*, the Δ*Moscs2#3* produced considerably fewer conidia and reduced approximately 60% in conidiation (Figures 3A,B).

In addition, part of the conidia produced by the Δ*Moscs2* mutant showed aberrant morphology (Figure 3C). The conidial length of the Δ*Moscs2* mutant was significantly shorter (Figure 3D), further examination by CFW staining showed that over 90% of conidia of the Δ*Moscs2* mutant showed abnormal morphology which had only one or no septum, while the conidia of the WT were pyriform and 85% possessed two septa (Figures 3C,D). These results indicated that the loss of *MoSCS2* affects conidiogenesis and conidial morphology.

### Deletion of *MoSCS2* impaired the pathogenicity of *Magnaporthe oryzae*

**Figure 4:**
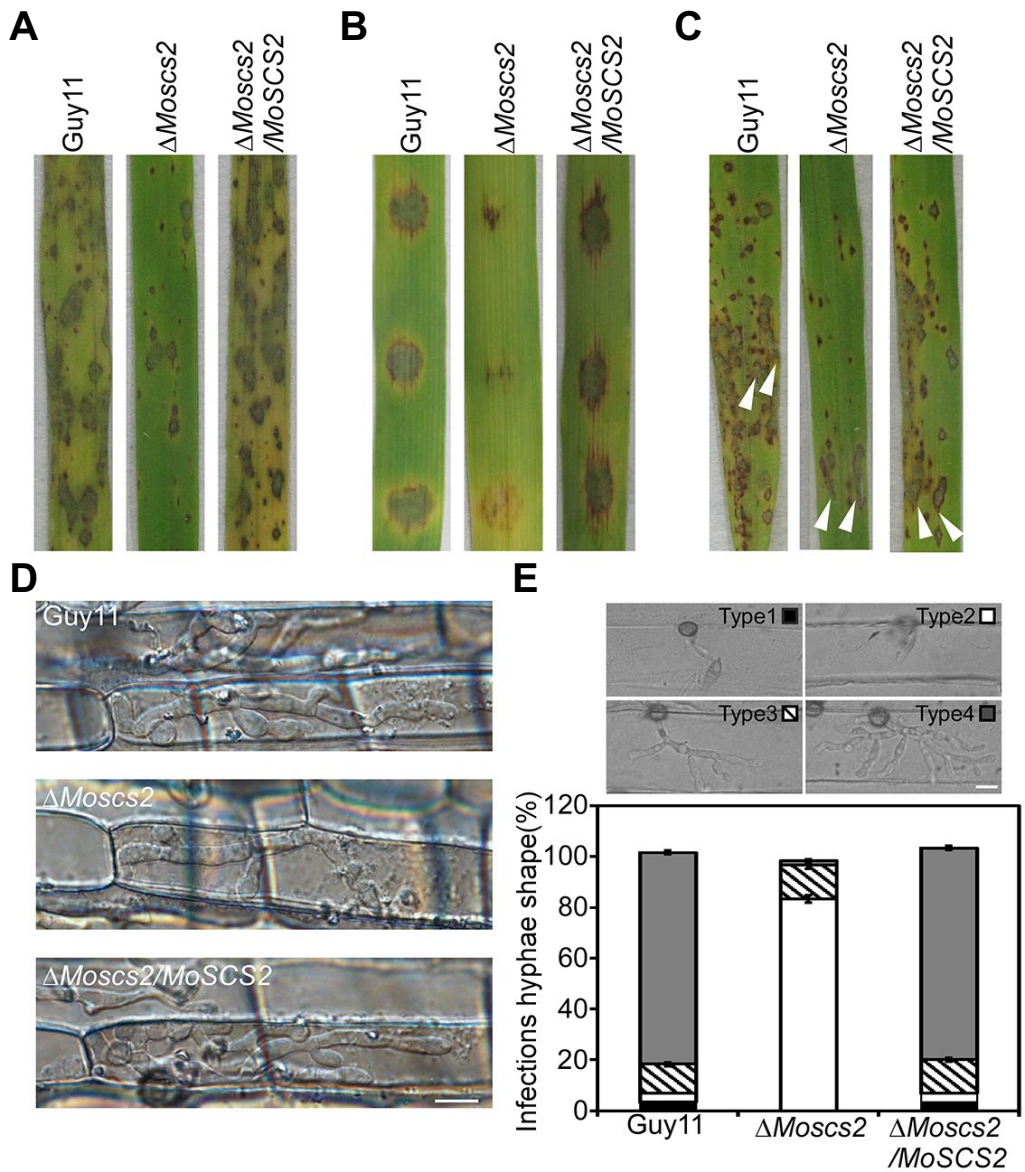
MoScs2 is crucial for the pathogenicity of *M. oryzae*. **(A)** Rice seedlings were sprayed with conidial suspension from the indicated strains and examined at 7 dpi. **(B)** Detached barley leaves were drop-inoculated with conidial suspensions and examined at 5 dpi. **(C)** Pathogenicity was tested by injection with conidia of the indicated strains. **(D)** Excised rice sheath from 4-week-old rice seedlings was inoculated with conidial suspensions. Infectious growth was observed at 1.5 dpi. **(E)** Statistical analysis for each type (type 1, no penetration; type 2, with penetration peg; type 3, with a single invasive hypha; type 4, with extensive hyphal growth) of infectious hyphal shape, for each tested strain; more than 30 infected sites were counted per replicate. Experiments were repeated with similar results. Scale bar = 10 μm.

To further test the role of *MoSCS2* in pathogenesis, conidial suspensions (5 × 10^4^ spores/ml) from the wild-type strain Guy11, the Δ*Moscs2* mutant and complemented strain were sprayed onto susceptible rice seedlings of CO-39. It was found that the Δ*Moscs2* mutant produced significantly fewer lesions than wild-type strains after 7 days of inoculation. Additionally, the lesions produced by Δ*Moscs2* mutant were also smaller and less expansive, in contrast to the fully expanded necrotic lesions produced by wild-type strain Guy11 and the complemented strain (Figure 4A). We obtained similar results from infection assays with seedlings of barley or by using the injection inoculation on rice (Figures 4B,C). The results demonstrate that *MoSCS2* plays a vital role in pathogenicity.

### A reduction in pathogenicity in Δ*Moscs2* mutant was attributed to defects in penetration and invasive hyphae growth

We inoculated rice leaf sheath and barley epidermal cells with the conidial suspensions. Following 36 h of incubation with rice leaf sheaths containing spore suspensions, invasive hyphae formed freely and expanded into neighboring cells in the wild type and the complementation strains. By contrast, the Δ*Moscs2* mutant showed restricted invasive hyphal growth, which was confined to the first plant cells (Figure 4D).

Furthermore, a similar result was observed when inoculations were done on barley epidermal cells. After incubation with spore suspensions for 36 h, compared with the WT and complemented strains, fewer invasive hyphae were observed in the Δ*Moscs2* mutant, and the branches are significantly reduced. We classify the invasive hyphae into 4 types (type 1, no penetration; type 2, with penetration peg; type 3, with a single invasive hypha; type 4, with extensive hyphal growth). In WT and the complemented strains, approximately 80% of cells displayed type 4, 15% showed type 3 and 5% showed type 2. Nonetheless, less than 20% of cells showed type 4 and type 3, and 80% showed type 1 and type 2 in the Δ*Moscs2* mutant (Figure 4E). Taken together, these results suggest that *MoSCS2* is required for appressorial penetration and invasive hyphal growth. Moreso, these data suggest that the limited invasive hyphal extension of the mutant may be the cause of a significant reduction in pathogenicity.

### *MoSCS2* is involved in the regulation of appressorium development, turgor pressure and host-produced reactive oxygen species (ROS) accumulation

**Figure 5:**
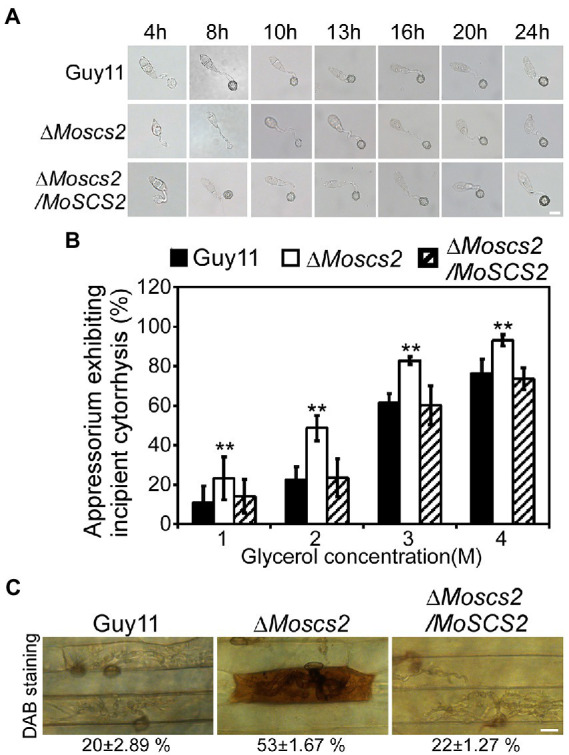
Effects of MoScs2 on appressorium development and turgor pressure of *M. oryzae*. **(A)** Appressorium formation were tested on artificial hydrophobic surface of the indicated strains at different time-points. **(B)** Collapsed appressoria were observed in mutant strain. For each glycerol concentration, at least 60 appressoria were observed and the numbers of collapsed appressoria were counted. Error bars represent SD of three replicates and asterisks represent a significant difference between Guy11 and the Δ*Moscs2* strain (Duncan’s new multiple range test, *p* < 0.01). **(C)** DAB-staining of the indicated strains-infected barley epidermis cells. Percentage of DAB-stained barley epidermis cells infected by the strains were shown at the bottom of the micrograph. Scale bar = 10 μm.

To further investigate whether the compromised pathogenicity of the Δ*Moscs2* mutant was related to the defects in appressorium development, spores of WT, the Δ*Moscs2* mutant and the complemented strains were inoculated onto artificial hydrophobic surfaces. After inoculation for 8 h, compared with the WT and complemented strains, the majority of the Δ*Moscs2* mutant produced unmelanized appressoria. The appressorium formation of the Δ*Moscs2* mutant appeared to be delayed, and the conidia germination rate was lower than that of the WT. However, the appressorium formation and maturation were similar in all the strains by 24 h (Figure 5A). These results indicated that deletion of *MoSCS2* gene delayed appressorium development, but did not affect the appressorium formation.

The huge appressorium turgor pressure is essential for *M. oryzae* to penetrate the plant epidermal cell and causes disease. To further clarify whether the deletion of *MoSCS2* gene affects the turgor pressure in appressorium and leads to attenuation of pathogenicity, we tested the appressorium turgor pressure by cytolysis assays. We treated appressoria with 1 M, 2 M, 3 M, and 4 M glycerol, respectively. Our results showed that the Δ*Moscs2* mutant displayed a higher collapsed rate than the WT and complemented strains. Moreover, the collapse rate of appressorium in the Δ*Moscs2* mutant was twice that of the others at 1 M and 2 M glycerol (Figure 5B), indicating MoScs2 is important for normal turgor of appressorium. Moreover, 3,3′-diaminobenzidine (DAB) staining assays of the penetrated plant cells showed the accumulation of ROS at the infection site of the Δ*Moscs2* mutant, but not the wild type and complemented strains (Figure 5C). These results indicated that deletion of *MoSCS2* affects appressorium development, turgor pressure and host-produced ROS accumulation.

### MoScs2 contribute to ER morphology

**Figure 6:**
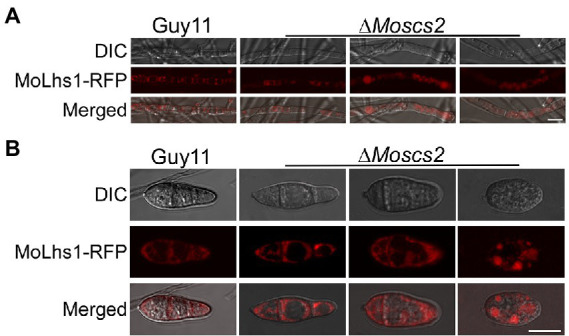
MoScs2 is involved in the regulation of ER morphology. ER marker protein MoLhs1-RFP was introduced into the indicated strain and hyphae **(A)** and conidia **(B)** of the resulting transformants were observed under an epifluorescence microscope. Scale bar = 10 μm.

We further determined whether the deletion of *MoSCS2* contributes to ER morphology by using Lhs1-RFP transgenic strains. Lhs1 is an endoplasmic reticulum protein, a RFP-labeled LHS1 was expressed in both WT and the Δ*Moscs2* mutant to monitor ER morphology. Compared with the WT, the ER morphology of about 56 ± 3.9% vegetative mycelium and 63 ± 3.6% conidia of the Δ*Moscs2* mutant was abnormal (Figures 6A,B), with a majority of the ER appearing to be invaginated or diffuse. This result indicated that the deletion of *MoSCS2* gene would affect the ER morphology.

### MoScs2 is involved in the regulation of ER stress response

**Figure 7:**
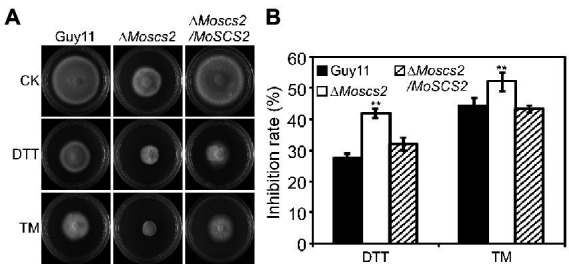
The Δ*Moscs2* mutant heightened sensitivity to ER stress. **(A)** Growth of Guy11, the Δ*Moscs2* mutant and complemented strains in CM media supplemented with TM or DTT. **(B)** Colony diameters were determined in each independent biological experiment after 7 days. Measurements of growth inhibition rate are relative to the growth rate of each untreated control. Error bars represent SD of three replicates and asterisks represent a significant difference between Guy11 and the Δ*Moscs2* strain (Duncan’s new multiple range test, *p* < 0.01).

Dithiothreitol (DTT) and tunicamycin (TM) are typical inducers that disturb ER homeostasis and induce ER stress. To investigate whether MoScs2 is important for the ER stress response, we test the sensitivity of the Δ*Moscs2* mutant strains towards DTT and TM. When exposed to 2.0 mM DTT, the Δ*Moscs2* mutant exhibited remarkably heightened sensitivity to DTT (Figure 7A). The deletion of both *MoSCS2* caused severe inhibitory hyphal growth (42% inhibition rate). Similarly, when exposed to 0.2 μg/ml TM, the Δ*Moscs2* mutant heightened significantly sensitivity compared with the WT and the complemented strain with a 50% inhibition rate (Figure 7B). These data suggest that *MoSCS2* is involved in the regulation of ER stress response.

### MoScs2 is involved in the regulation of cell wall stress responses

**Figure 8:**
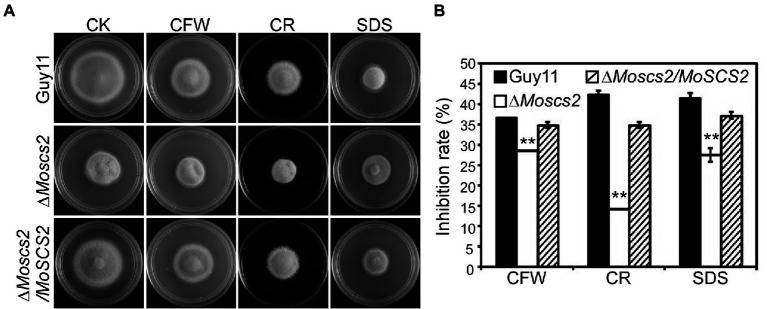
**(A)**Effects of MoScs2 on cell wall integrity of *M. oryzae.* Growth of Guy11, the Δ*Moscs2* mutant and complemented strains in CM media supplemented with CFW, CR or SDS. **(B)** Colony diameters were determined in each independent biological experiment after 7 days. Measurements of growth inhibition rate are relative to the growth rate of each untreated control. Error bars represent SD of three replicates and asterisks represent a significant difference between Guy11 and the Δ*Moscs2* strain (Duncan’s new multiple range test, *p* < 0.01).

To investigate the contribution of *MoSCS2* genes in cell wall stress responses, the vegetative growth of all strains was monitored and measured on CM medium with the addition of cell wall stressors Calcofluor white (CFW), Sodium Dodecyl Sulfate (SDS), and Congo Red (CR). Following 7 days post-inoculation, our results indicated that the inhibition rate of the three cell wall stressors against the Δ*Moscs2* mutant was lower than that of the WT and the complemented strain (Figures 8A,B). The above results indicated that MoScs2 is involved in the regulation of cell wall stress responses of *M. oryzae.*

### Functional characterization of MoScs2 transmembrane domain

**Figure 9:**
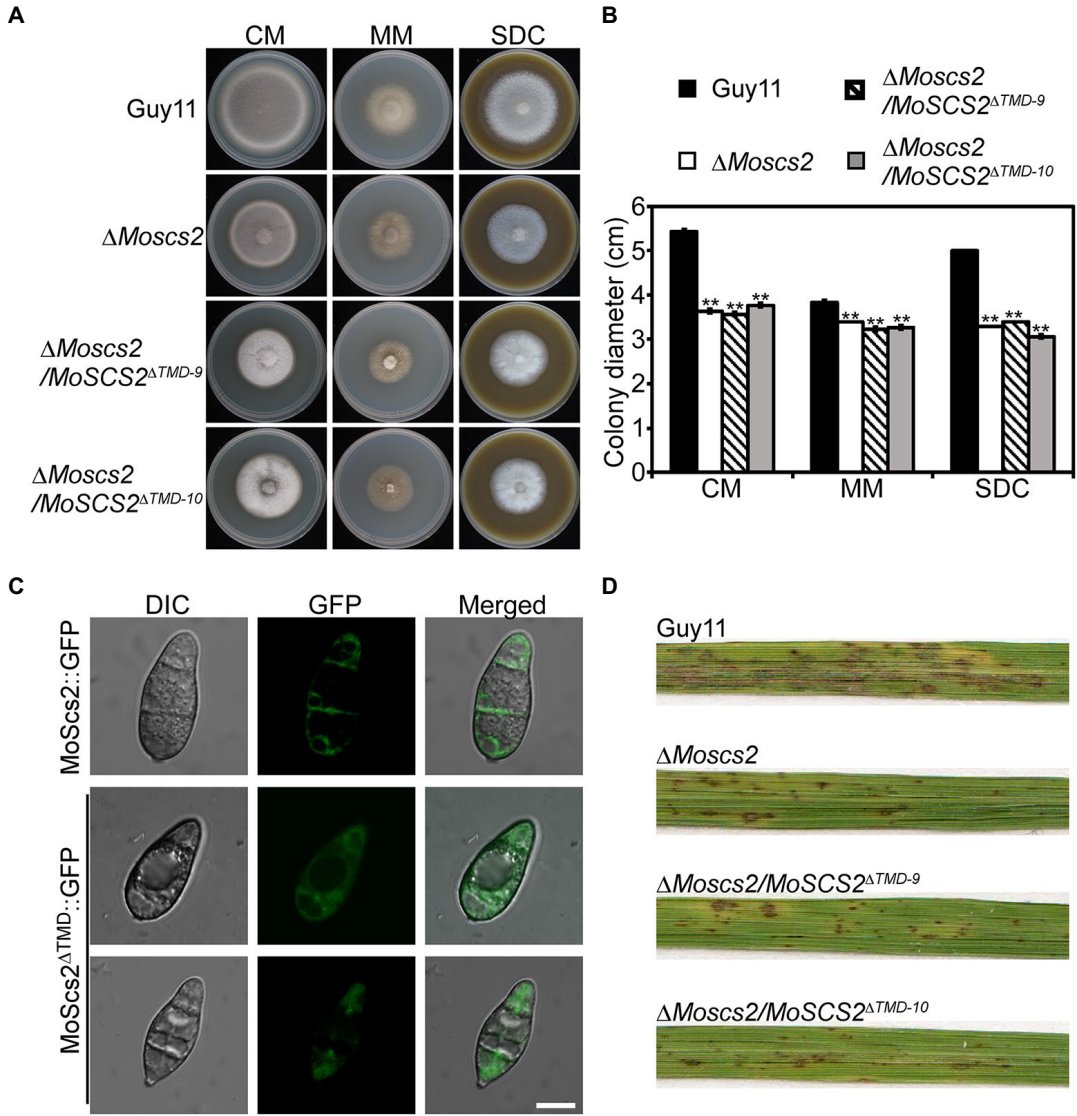
Functions of TMD domain of MoScs2 in *M. oryzae.* **(A)** The wild-type (Guy11), Δ*Moscs2* mutant, and Δ*Moscs2/MoSCS2^Δ^^TMD^* strains were inoculated on CM, MM, and SDC media and cultured at 28°C in the dark for 7 days and then photographed. **(B)** Statistical analyses of the colony diameter of Guy11, the Δ*Moscs2* mutant, Δ*Moscs2/MoSCS2^Δ^^TMD^* strains on different media. Error bars represent the standard deviations; asterisks denote statistical significances (*p* < 0.01). **(C)** Subcellular localization of the MoScs2^Δ^^TMD^-GFP fusion proteins. Conidia of the transformant expressing MoScs2-GFP or MoScs2^Δ^^TMD^-GFP were examined by differential interference contrast (DIC) and epifluorescence microscopy. Scale bar = 10 μm. **(D)** Two-week-old rice seedlings were sprayed with conidial suspensions of the wild-type (Guy11), Δ*Moscs2* mutant, and Δ*Moscs2/MoSCS2^Δ^^TMD^* strains. Diseased leaves were photographed 7 days after inoculation.

To clarify the function of a transmembrane domain (TMD) of *MoSCS2* during vegetative growth, domain deletion constructs were fused with GFP to generate the MoScs2^Δ^*^TMD^*-GFP (loss of the transmembrane domain) which were transformed into the Δ*Moscs2* mutant. The resulting transformants (Δ*Moscs2*/*MoSCS2*^Δ^*^TMD^*) were evaluated for GFP signals and analyzed. The wild-type (Guy11), Δ*Moscs2* mutant, and Δ*Moscs2*/*MoSCS2*^Δ^*^TMD^* strains were inoculated on CM, MM, and SDC media. After 7 days, compared with the wild-type strain, the Δ*Moscs2*/*MoSCS2*^Δ^*^TMD^* showed a reduced hyphal growth rate similar to that of the Δ*Moscs2* mutant (Figures 9A,B). We also observed the localization of the transmembrane domain deletion transformant. Compared with the MoScs2-GFP strain, the GFP signal of the Δ*Moscs2*/*MoSCS2*^Δ^*^TMD^* mutant was diffused in the conidia. We further test the pathogenicity of these mutants on rice and found that the Δ*Moscs2*/*MoSCS2*^Δ^*^TMD^* mutants displayed pathogenic defects similar to that of the Δ*Moscs2* mutant (Figure 9D). The above results indicate that the TMD of *MoSCS2* is pivotal for the growth, pathogenicity and subcellular localization in *M. oryzae* (Figure 9C).

## Discussion

In *S. cerevisiae*, the absence of Scs2 protein will lead to the separation of ER and plasma membrane, resulting in the ER dysfunction, but its function remains unkonwn in rice blast fungus or even filamentous fungus. In this study, we focused on the characteristics of *MoSCS2* in *M. oryzae* and its effect on the vegetative growth, asexual reproduction, and pathogenicity of *M. oryzae*. To elucidate the function of *MoSCS2* in *M. oryzae*, *MoSCS2* gene was deleted by homologous recombination, and the morphological characterization and pathogenicity of wild-type strain Guy11, *MoSCS2* deletion mutant and its complementary transformant were analyzed.

In yeast cells, Scs2/Scs22 localizes to both the nuclear and cytoplasmic ER structures in addition to the cER (Loewen et al., 2007). After the deletion of Scs2/Scs22, the cER was significantly reduced and PI4P accumulation level was higher (Manford et al., 2012). What we found in *M. oryzae* is that MoScs2 was localized in the ER. And compared to wild-type strain Guy11, the growth rate of Δ*Moscs2* mutant is significantly slower, and the asexual reproduction ability of the mutant is significantly reduced. Further experiments showed that the deletion of *MoSCS2* also affected the septum formation of the conidia, and delayed the appressorium development. Plant leaves infection assays showed that knockout of *MoSCS2* significantly inhibited the expansion of the invasive hyphae in cells. This result may reflect the important role of MoScs2 during invasive growth. Moreover, the appressorium of Δ*Moscs2* mutant could not form huge turgor pressure to penetrate the host epidermal cell wall, and invasive hyphal growth was limited in host cells, probably ultimately leading to the pathogenic defects.

ER is known to be the main intracellular organelle responsible for protein synthesis and secretion. Based on the phenotype of Δ*Moscs2* mutant, we speculated that the impaired ER morphology of the mutant may lead to ER dysfunction. To test this hypothesis, we treated Δ*Moscs2* mutant with DTT and TM which could disturb ER homeostasis and induce ER stress, respectively. Then we found that the mutant was more sensitive to the ER pressure inducers. The above results all confirmed that the ER homeostasis was compromised in the Δ*Moscs2* mutant. In addition, there is evidence that ROS are critical components during the host-pathogen interaction, and play key roles in rice against *M. oryzae* (Kou et al., 2019). According to our research, DAB staining assays of the penetrated plant cells showed the accumulation of ROS at the infection site of the Δ*Moscs2* mutant. This result suggests that *MOSCS2* is involved in regulating host-driven ROS scavenging during infection. The rice blast fungus secretes a large number of effectors into rice cells to suppress plant immunity. We speculate that the ER dysfunction in the Δ*Moscs2* mutant may lead to a defective secretory efficiency and then disturb the effector secretion to suppress immunity. It would be interesting to determine the secretion of known effector proteins in the Δ*Moscs2* mutant in future research.

Scs2 contains a single transmembrane domain (TMD) and a cytoplasmic MSP domain (Manford et al., 2012). Moreover, the mutant Scs2 protein lacking its TMD localized in the nucleus rather than the ER (Manford et al., 2012). According to our results, the pathogenicity of the Δ*Moscs2* mutant reduced after the function of the ER was impaired. To clarify the cause, we knock out the TMD of *MoSCS2* in *M. oryzae*. Based on the phenotype analysis we found that the Δ*Moscs2*/*MoSCS2*^Δ^*™^D^* mutant was unable to restore the defective phenotype and the protein localization. These results suggest that the TMD domain plays an important role in the function of *MoSCS2*.

In conclusion, our study illustrates that MoScs2 plays an important role in the growth, asexual development, conidia morphogenesis, appressorium formation and pathogenicity of *M. oryzae*, but its specific pathogenic mechanism needs further study.

## Data availability statement

The original contributions presented in the study are included in the article/Supplementary material; further inquiries can be directed to the corresponding authors.

## Author contributions

ZW and WT designed this study. JZ, XC, ZY, HX, and SW performed experiments and analyzed all data. JZ, WT, and ZW wrote the initial manuscript. All authors contributed to the article and approved the submitted version.

## Funding

This research was funded by the National Natural Science Foundation of China (No. U1805232), the Special Fund Project for Science and Technology Innovation of Fujian Agriculture and Forestry University (No. CXZX2020014A), and the Program of Fujian Key Laboratory for Monitoring and Integrated Management of Crop Pests, Fuzhou 350013, China (No. MIMCP-202101).

## Conflict of interest

The authors declare that the research was conducted in the absence of any commercial or financial relationships that could be construed as a potential conflict of interest.

## Publisher’s note

All claims expressed in this article are solely those of the authors and do not necessarily represent those of their affiliated organizations, or those of the publisher, the editors and the reviewers. Any product that may be evaluated in this article, or claim that may be made by its manufacturer, is not guaranteed or endorsed by the publisher.

## Footnotes

^1^ http://fungidb.org/fungidb/
