# Supplementary material for: Endoplasmic reticulum membrane protein MoScs2 is important for asexual development and pathogenesis of Magnaporthe oryzae
Source: Front Microbiol. 2022 Aug 4;13:906784. doi: 10.3389/fmicb.2022.906784 (PMC9386004; doi:10.3389/fmicb.2022.906784)
Supplement: Supplementary file 1 [file Table_1.DOCX]

Supplementary Material

# Supplementary Figures and Tables

## Supplementary Figures


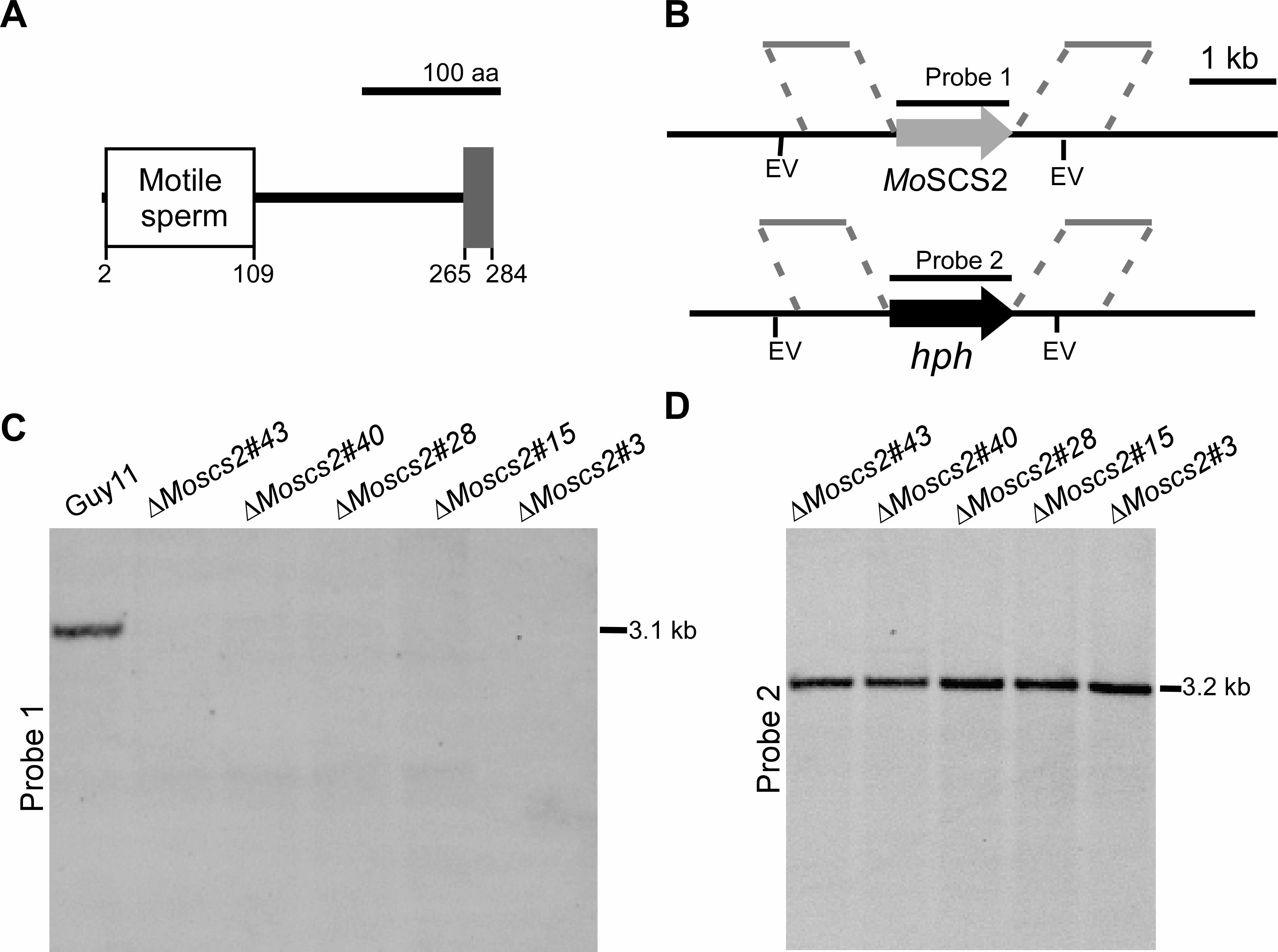


Figure S1. Analysis of MoScs2 protein conserved domains and southern blot analysis of *MoSCS2* deletion. (A) Prediction of domains of MoScs2 using the SMART website (<http://smart.embl-heidelberg.de/smart/set_mode.cgi?NORMAL=1>). (B) Strategy of knocking out *MoSCS2* gene in *M. oryzae* genome. (C and D) DNA gel blot analysis of genomic DNA of Guy11, Δ*Moscs2* mutants digested with *Eco*R V(EV) and probed with a gene-specific probe (probe 1) and hygromycin phosphotransferase (HPH) probe (probe 2). The estimated sizes of each band are labeled at right (kb).

## Supplementary Table

Table S1 Primers used in this study

| **Name** | **Sequence (5’-3’)** | **Remark** |
| --- | --- | --- |
| SCS2 f1  SCS2 f2  SCS2 f3  SCS2 f4  SCS2 koF  SCS2 koR  SCS2 CF  SCS2 CR  HYG F  HYG R | TACGTACCTGTGATTAGCG  CATTCATTGTTGACCTCCACTAGCTCCATGTCACCGGCGTGGTGGT  GCAAAGGAATAGAGTAGATGCCGACCGAGGGAAACTCAGTTATGC  ACGAGATTGCGAGCGAGT  CCTCTGGGCGAATAATTTGA  TCTCCTTGACCTGCTCCACT  ACTCACTATAGGGCGAATTGGGTACTCAAATTGGTTTGACTGCGTCATGTTGATGG  CACCACCCCGGTGAACAGCTCCTCGCCCTTGCTCACGAAGAAGAAATAGGCAAGCAG  GGCTTGGCTGGAGCTAGTGGAGGTCAA  CGGTCGGCATCTACTCTATTCCTTTG | amplify *MoSCS2* 5’ flank sequence  amplify *MoSCS2* 5’ flank sequence  amplify *MoSCS2* 3’ flank sequence  amplify *MoSCS2* 3’ flank sequence  amplify *MoSCS2* probe sequence  amplify *MoSCS2* probe sequence  *MoSCS2* complementation  *MoSCS2* complementation  amplify *HPH* probe sequence  amplify *HPH* probe sequence |
| SCS2 TR | CACCACCCCGGTGAACAGCTCCTCGCCCTTGCTCACGCCCTGGGGCGCCTGCTGAGC | *MoSCS2*^ΔTMD^ construction |
